# Contextual cueing: implicit memory of tactile context facilitates tactile search

**DOI:** 10.3758/s13414-015-0848-y

**Published:** 2015-03-04

**Authors:** Leonardo Assumpção, Zhuanghua Shi, Xuelian Zang, Hermann J. Müller, Thomas Geyer

**Affiliations:** 1 Department Psychologie, Lehrstuhl für Allgemeine und Experimentelle Psychologie, Ludwig-Maximilians-Universität München, Leopoldstraße 13, 80802 München, Germany; 2 School of Psychology, Birkbeck College, University of London, London, UK

**Keywords:** Contextual cueing, Implicit memory, Attention, Tactile search, Haptics, Touch

## Abstract

In visual search, participants detect and subsequently discriminate targets more rapidly when these are embedded in repeatedly encountered distractor arrangements, an effect termed *contextual cueing* (Chun & Jiang *Cognitive Psychology, 36*, 28–71, 1998). However, whereas previous studies had explored contextual cueing exclusively in *visual search*, in the present study we examined the effect in *tactile search* using a novel tactile search paradigm. Participants were equipped with vibrotactile stimulators attached to four fingers on each hand. A given search array consisted of four stimuli (i.e., two items presented to each hand), with the target being an odd-one-out feature singleton that differed in frequency (Exps. 1 and 2) or waveform (Exp. 3) from the distractor elements. Participants performed a localization (Exps. 1 and 2) or discrimination (Exp. 3) task, delivering their responses via foot pedals. In all three experiments, reaction times were faster when the arrangement of distractor fingers predicted the target finger. Furthermore, participants were unable to explicitly discriminate repeated from nonrepeated tactile configurations (Exps. 2 and 3). This indicates that the tactile modality can mediate the formation of configural representations and use these representations to guide tactile search.

Humans experience a myriad of events at any given time, presenting an excessive load of information to the brain. However, most events or objects do not occur in isolation; rather, they are embedded in larger, structured environments. Previous work has shown that environmental regularities are permanently retained and facilitate visual perception. For instance, in a seminal study by Palmer (1975; see also Biederman, 1972; Biederman, Mezzanotte, & Rabinowitz, 1982; Chun, 2000; Hollingworth, 1998), participants were presented with a scene context (e.g., a kitchen counter) followed by a brief presentation of a target that was either context-appropriate (e.g., a loaf of bread), context-inappropriate but similar in shape to the appropriate object (e.g., a mailbox), or completely context-inappropriate (e.g., a drum). In a subsequent naming task, participants showed higher performance accuracy in the context-appropriate than in the two context-inappropriate conditions. Palmer concluded that *visual object recognition* is modulated by scene context.

The beneficial effect of environmental information on *visual selective attention* was further elucidated by Chun and Jiang (1998), by means of their contextual-cueing paradigm. In this task, participants have to detect and subsequently discriminate the orientation (left vs. right) of a target “T” embedded in a set of distractor “L”s. Unbeknownst to participants, half of the trials contain repeated and the other half nonrepeated target–distractor spatial arrangements. In the repeated—*old*—condition, both the target and the distractors are presented at identical display locations across trials. By contrast, in the nonrepeated—*new*—condition, only the targets (but not the distractors) appear at identical locations (by keeping target locations constant in the old and new displays, one can equate target location repetition effects between the two types of displays and thus isolate the effect of context on reaction time—RT—performance). Chun and Jiang found that participants were faster at detecting the target in old than in new displays, an effect referred to as *contextual cueing.* Interestingly, when participants were asked to discriminate the repeated from the nonrepeated displays, explicit recognition was only at chance level. This dissociation in direct (recognition) and indirect (RT) measures led Chun and Jiang to surmise that contextual cueing is supported by an implicit memory system.

In recent years, Chun and Jiang’s (1998) basic findings and paradigm have inspired numerous studies. For example, van Asselen and Castelo-Branco (2009) showed that contextual cueing was still obtained in a test session when the training and test sessions were separated by 10 days. Geyer, Müller, Assumpção, and Gais (2013) found that even a short nap relative to an equivalent period of controlled rest separating the learning and test sessions (on the same day) was sufficient to enhance contextual cueing. Other investigations have demonstrated that, rather than relying on the entire distractor context, contextual cueing is supported by memory for individual target–distractor (paired) associations formed particularly in the vicinity of the target (Brady & Chun, 2007; Jiang & Wagner, 2004; Shi, Zang, Jia, Geyer, & Müller, 2013), or amongst distractors sharing the target’s color (Conci, Müller, & von Mühlenen, 2013; Geyer, Shi, & Müller, 2010). Additional work has shown that contextual learning (i.e., the acquisition of contextual memory) and expression (i.e., the retrieval of contextual memory) are separate processes (Chaumon, Schwartz, & Tallon-Baudry, 2009) and that an additional (spatial working memory) task interferes with the retrieval, but not the learning, of contextual memory representations (Annac et al., 2013).

Taken together, the by now extensive body of studies on contextual cueing in *visual* search has contributed substantially to our understanding of the processes underlying *implicit spatial learning*. Although sensory modalities other than vision have received considerable interest in recent years (e.g., Klatzky, Lippa, Loomis, & Golledge, 2002, 2003; Yamamoto & Shelton, 2009), little is known about the roles of these senses for implicit context learning. Concerning the haptic/tactile sense, a growing, although still modest, number of studies have revealed intricate processing capabilities of this modality. For example, it has been demonstrated that features such as material dimensions and abrupt surface discontinuities are likely to produce low search function slopes, suggesting a parallel search, whereas orientation and 3-D surface contours are likely to yield somewhat steep slopes, suggesting serial search (Lederman & Klatzky, 1997). More recently, a study focusing on a manual 3-D search task demonstrated that saliency was an important factor in determining what parts of the hand and what strategies would be used to contact the target, suggesting that nonsalient conditions made participants more likely to engage the thumb in a serial strategy, whereas in salient target conditions, parallel strategies such as grasping and shuffling of the items in the hand were applied (van Polanen, Bergmann Tiest, & Kappers, 2014). With respect to spatial learning and representations, it has been claimed that the haptic sense can facilitate (the updating of) visuospatial representations (Shelton & McNamara, 2001), or even that participants are able to form entirely new spatial representations in an explicit learning task on the basis of haptic information alone (Pasqualotto, Finucane, & Newell, 2005). However, whether the tactile sense is capable of forming its own *implicit spatial representation* and the extent to which such representations can be used for attentional guidance remain open questions. This is the issue that we investigated in the present study.

Hitherto, to our knowledge, only one study, by Nabeta, Ono, and Kawahara (2003), has attempted to investigate haptic contextual cueing. Nabeta et al. found facilitation of RTs for old relative to new *haptic* arrangements when the old (haptic) arrangements were learned in a preceding visual search task (i.e., the same arrangements were used in visual and haptic tasks). However, it was not clear whether this haptic contextual-cueing effect was driven by haptic or visual representations. That is, they could not rule out that in the haptic task, participants may have continued to operate an essentially visual strategy (see, e.g., Lederman, Klatzky, Chataway, & Summers, 1990, for the effects of visual imagery on recognition performance in a haptic discrimination task). For example, participants may have registered the haptically sensed stimuli in a visuospatial representation maintained in working memory, and it may have been this representation that, when critical (context) stimuli had been sampled and recorded, triggered the matching visual context information stored in long-term memory, thus guiding haptic search to the (visually represented) target location. Note that Nabeta et al.’s participants did not see the haptic displays, so search could ultimately only be based on some actively built-up and maintained spatial working memory representation. In other words, the observed haptic contextual cueing might well have been visually mediated. Furthermore, Nabeta et al. did not address the fundamental question: that is, whether contextual regularities can also be acquired in—rather than in one way or another “transferred” to—the haptic modality. The present study was designed to examine this question.

Participants were tested in an exclusively tactile search task, which adopted Chun and Jiang’s (1998) original approach, with half of the trials containing old and the other half new arrangements. In Experiment 1, we investigated contextual cueing in tactile search, whereas in Experiment 2 we assessed participants’ explicit knowledge of repeated tactile arrangements. In Experiment 3, we introduced a discrimination task in order to dissociate the contextual cueing of target selection from the contextual cueing of response selection.

## Experiment 1

### Method

#### Participants

Nine naïve participants (eight female, one male, eight right-handed; age range 24 to 41 years) took part in this experiment for either course credit or €8.00/h. All of the participants reported normal tactile perception and no history of somatosensory disorders. Participants gave informed consent prior to performing the experiment, which was approved by the ethics committee of the Department of Psychology at LMU Munich, in accordance with the Code of Ethics of the World Medical Association (Declaration of Helsinki).

#### Apparatus and stimuli

**Fig. 1:**
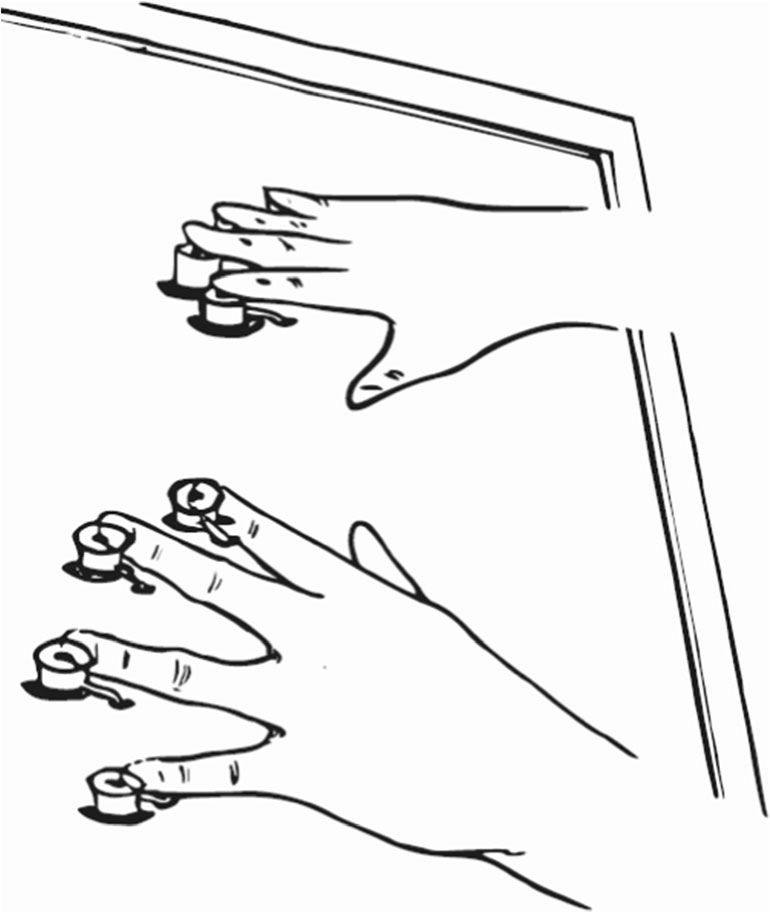
Illustration of the experimental setup. Participants placed their fingers (except the thumbs) on eight solenoids delivering tactile stimulation. The solenoids are indicated by the rings in the figure. In Experiments 1 and 2, participants indicated the location of a feature-singleton target, defined by a different frequency relative to the distractors, as being delivered to a left- or a right-hand finger by pressing the corresponding (left or, respectively, right) foot pedal. In Experiment 3, participants indicated the target identity using the appropriate foot response, regardless of the hand or finger stimulated

The vibrotactile stimuli, 100- and 30-Hz vibrations, were generated by eight solenoid actuators that activated lodged cylinder metal tips when the solenoid coils were magnetized (Heijo Box, Heijo Research Electronics, UK; see Fig. 1). The maximum finger contact area was about 2–4 mm. The eight actuators, connected to a “standard” PC via parallel port, were controlled by a purpose-written MATLAB program in combination with the Psychophysics Toolbox (Brainard, 1997; Pelli, 1997). Participants’ responses were recorded via foot pedals. In the practice phase, visual information such as instructions, fixation cross, and response feedback was video-projected onto a semitransparent Plexiglas table (size, 70 × 60 cm; height, 84 cm) by a projector (Sharp XR-32X-L), and was therefore available for the participants to monitor. In the experimental phase, a blindfold was used to prevent participants from seeing the tactile arrangements (and thus to avoid visual learning of the tactile arrays). Furthermore, the vibrotactile stimulations were masked by white noise (1000 Hz, ~65 dBA, 3,000 ms or until response execution) delivered via cushioned ear shell headphones (Philips SHL4000, 30-mm speaker drive). This was again done in an attempt to rule out confounding factors in the determination of participants’ performance, such as auditory learning of the tactile arrays: Note that different vibrotactile stimulations generate different tones, thus potentially offering an additional, auditory source of information for configural learning.

#### Procedure

Following written and verbal instructions, participants were equipped with headphones, and, once they were comfortably seated on a chair with their forearms on the table, gently placed their fingertips (except the thumbs) on the stimulators. Prior to the practice session, the positions of the stimulators were adjusted to fit the participant’s fingers. In order to maximize comfort, participants chose whether or not they wanted to use a cushion as a wrist-rest throughout the experiment.

#### Practice session

Each trial of the practice session started with a foot press and included stimulations delivered to seven distractor fingers vibrating at 100 Hz and one target finger vibrating at 30 Hz. All stimulators vibrated until a response was made or up to 3,000 ms, whichever came first. In doing so, the target was presented four times at any of the eight fingers, yielding 32 practice trials in total. Note that, given the limited number of tactile configurations available for the experimental session (see below), the practice session used stimulations of all eight fingers. The idea was to familiarize participants with the tactile search task—that is, target-versus-distractor discrimination in general—rather than the learning of specific tactile arrangements. Participants’ task was to localize the target, as quickly as possible, on the left or the right hand by pressing the spatially corresponding foot pedal. Following participants’ responses, accuracy feedback was provided by presenting the words “correct” or “wrong” on the Plexiglas plate (duration: 1,000 ms). Participants were instructed to monitor this feedback and use it to improve their tactile search performance. Thus, emphasis was placed on both response speed and accuracy.

#### Experimental session

Following the instructions, participants started the experimental session by a foot press. Both the tactile vibration and white auditory noise were presented simultaneously until a response was made or up to 3,000 ms. Importantly, unlike in the practice session, in the experimental session participants were wearing a blindfold so that they did not see their fingers, thus preventing concomitant visual learning of the repeated tactile arrangements. Participants were asked to respond to the target side (either the left or the right) as quickly and accurately as possible by pressing the corresponding foot pedal. The next trial was automatically initiated following an intertrial interval of 1,000 ms.

#### Design

**Fig. 2:**
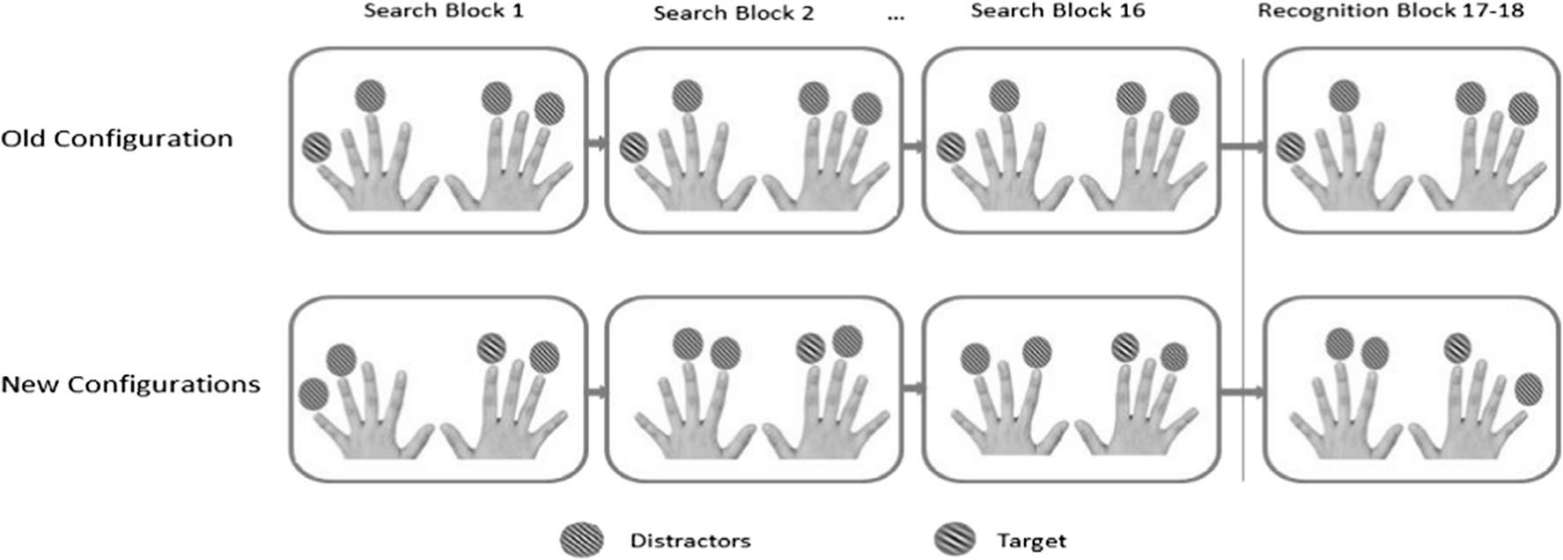
Schematic figure displaying the distribution of items in old and new configurations across search epochs (and the recognition task, for Exps. 2 and 3). In old configurations, the target location is constant and paired with constant distractor locations; in new configurations, by contrast, only the target location is held constant across repetitions

The design of Experiments 1–3 was adapted from that of Chun and Jiang (1998). On each trial of the experimental session, the tactile configuration consisted of stimulations of one target and three distractor fingers. To balance the vibrotactile stimulations between the two hands, a given tactile configuration always involved one hand with two distractors and the other hand with one distractor and one target (see Fig. 2). A set of four old configurations was randomly generated for each participant. For these old configurations, the relationship between the target and distractors was kept constant throughout the entire experiment (a block consisted of a set of four old plus four new configurations). The new configurations, by contrast, were newly generated in each block by distributing the three distractors anew across the remaining fingers of each hand on each new trial. Importantly, in the new condition, too, the four target fingers were held constant throughout the experiment. Thus, four fingers were used for targets in the old configurations (two fingers on each hand), and four fingers for the new configurations (again, two fingers on each hand). In doing so, participants had no bias to search for a target at specific fingers, since each finger was equally likely to contain a target. Performance gains in the old condition could therefore only be attributed to the effects of repeated tactile arrangements, rather than repeated absolute target locations. The latter were equated across the old and new tactile conditions. The experiment consisted of 128 trials, divided into 16 blocks. At the end of every second block, the white noise was interrupted, followed by the presentation of a brief double beep (2 × 200 ms, 1000 Hz, ~72 dBA; separated by an 800-ms silent interval), indicating that the participants could take a short break and resume the experiment (by a foot press) whenever they were ready to continue. The entire experimental session lasted about 30 min.

### Results

In order to increase statistical power, the data of two consecutive blocks were pooled together into one epoch (see Chun & Jiang, 1998), resulting in eight experimental epochs. For RTs, trials on which participants made an erroneous response or RTs were below 200 or above 3,000 ms (i.e., when no response was made) were excluded from the analysis (overall, 10.5 % of trials). The error and RT data were examined in repeated measures analyses of variance (ANOVAs), with any effects Greenhouse–Geisser corrected when sphericity was violated.

#### RT performance

**Fig. 3:**
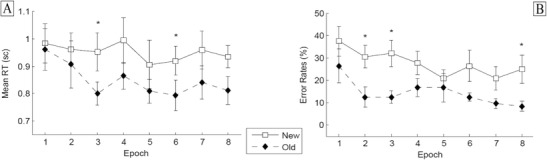
Experiment 1: **a** Mean response times across epochs for old and new configurations, with error bars representing within-participants standard errors of the means (Cousineau, 2005). **b** Mean error rates across epochs, shown separately for old and new configurations, with error bars representing standard errors of the means. ^*^ *p* < .05

A 2 × 8 factorial repeated measures ANOVA on the RTs revealed a significant main effect of configuration, *F*(1, 8) = 8.29, *p* < .05, *η*_p_^2^ = .509: Targets embedded in old configurations were detected significantly faster than those embedded in new configurations (849 vs. 952 ms), indicative of a tactile contextual-cueing effect (of 103 ms).^1^ Furthermore, RTs were relatively constant across experimental epochs [nonsignificant effect of epoch: *F*(7, 56) = 0.469, *p* = .673]. Although the Configuration × Epoch interaction was nonsignificant, *F*(7, 56) = 0.737, *p* = .642, additional *t* tests showed no difference between the RTs for old and new configurations in Epochs 1 and 2 (both *p*s > .2; see also Fig. 3). From this, one can conclude that reliable tactile contextual cueing developed over the course of the tactile search task.

#### Error analysis

A 2 × 8 factorial repeated measures ANOVA on the error rates with Configuration (old vs. new) and Epoch (1–8) as factors failed to reveal a significant effect of configuration, *F*(1, 8) = 3.85, *p* = .08, *η*_p_^2^ = .325. Because errors made in new configurations (13.80 %) were almost twice as high as those made in old configurations (7.20 %), we ran an additional (two-tailed) *t* test comparing the RTs between “old” and “new” response error trials in order to identify any possible speed–accuracy trade-off in the data. This test failed to reveal a significant effect of configuration, *t*(8) = 1.24, *p* = .247. Furthermore, the ANOVA revealed the effect of epoch to be significant, *F*(7, 56) = 2.31, *p* < .05, *η*_p_^2^ = .225, reflecting a decrease in the number of errors as the experiment progressed. The interaction between configuration and epoch did not reach statistical significance, *F*(1, 7) = 0.569, *p* > .778.

### Discussion

Experiment 1 employed a tactile search task in order to test whether tactile spatial context can be learned under exclusively tactile search conditions. The results provide clear evidence for this hypothesis. First, RTs were faster in old than in new tactile arrangements, an effect that became reliable after three epochs of learning (i.e., after five or six repetitions of each tactile configuration). Second, fewer response errors were made in old than in new tactile configurations (although this effect was nonsignificant), and the higher error rate in the latter condition was not due to a speed–accuracy trade-off. Nevertheless, response errors in both conditions decreased as the experiment progressed, as we observed in the main effect of epoch on response accuracy. Altogether, Experiment 1 provided evidence for context-dependent tactile learning, reflected by faster RTs in old than in new tactile arrangements and (numerically) fewer response errors to old arrangements. Context-independent procedural learning was also observed, reflected by a general reduction of error rates across experimental epochs.

A highly debatable claim in *visual contextual cueing* is whether the effect is supported by implicit memory (for a critical discussion, see, e.g., Schlagbauer, Müller, Zehetleitner, & Geyer, 2012, or Smyth & Shanks, 2008). Although Experiment 1 provided clear support for contextual cueing of tactile search, it left open the question of the implicit nature of the tactile contextual-cueing effect. To address this issue, in Experiment 2 we introduced a recognition test at the end of the experimental session to examine whether participants had awareness of the repeated tactile configurations.

## Experiment 2

### Method

Experiment 2 was a close replication of Experiment 1, with the following exceptions. In addition to implementing a recognition test, in Experiment 2 we implemented a more conservative practice regime, with the aim of reducing the relatively high rates of response errors made by participants in Experiment 1 (new configurations, 13.80 %; old configurations, 7.20 %). To this end, in Experiment 2 participants were informed that they would proceed from the practice to the experimental session only after having attained a minimum of 80 % correct responses in the practice session.

#### Participants

Fourteen new participants took part in Experiment 2 (seven female, seven male, 13 right-handed; age range 19 to 34 years). The criteria for participation, payments, and ethical guidelines were the same as in Experiment 1. Two participants were excluded because they showed unusually large contextual-cueing effects already in the first experimental epoch. A post-hoc analysis revealed that for these two participants, just by chance, the target fingers in old and new configurations were chosen in such a way that they were symmetrically allocated across hands, with old targets being presented at Fingers 2 and 4 and new targets at Fingers 1 and 3 of the left and right hands, respectively. This may have fostered the coupling of targets with specific (old, new) distractor arrangements. The percentage of trials excluded due to incorrect responses was 6.70 %. Outliers occurred in 0.06 % of all trials (i.e., RTs below 200 and above 3,000 ms).

#### Procedure

For the practice session, in addition to visual feedback, incorrect responses triggered an “error warning” beep (2500 Hz, ~85 dBA, 900 ms), followed by a silent intertrial interval of 2,000 or 2,500 ms. Furthermore, overall accuracy feedback was provided visually by displaying to participants their mean correct response rate after every second practice block (of 16 trials). Participants were asked to aim for a minimum of 80 % correct in at least three consecutive practice blocks. The experimental session was similar to that of Experiment 1, except for the use of “error warning” beeps following response errors. The entire experimental procedure lasted about 30–40 min.

#### Recognition task

At the end of the experimental session, participants performed a “yes–no” recognition task, meant to assess their explicit knowledge of the repeated tactile configurations. The recognition test consisted of 16 trials: 4 × 2 old and 4 × 2 new configurations, presented in randomized order, with the exception that a given display was never shown repeatedly on two consecutive trials. Because each old configuration was presented twice, each new configuration was also presented twice in order to equate the repetition effects across the two types of configurations.

### Results

#### RT performance

**Fig. 4:**
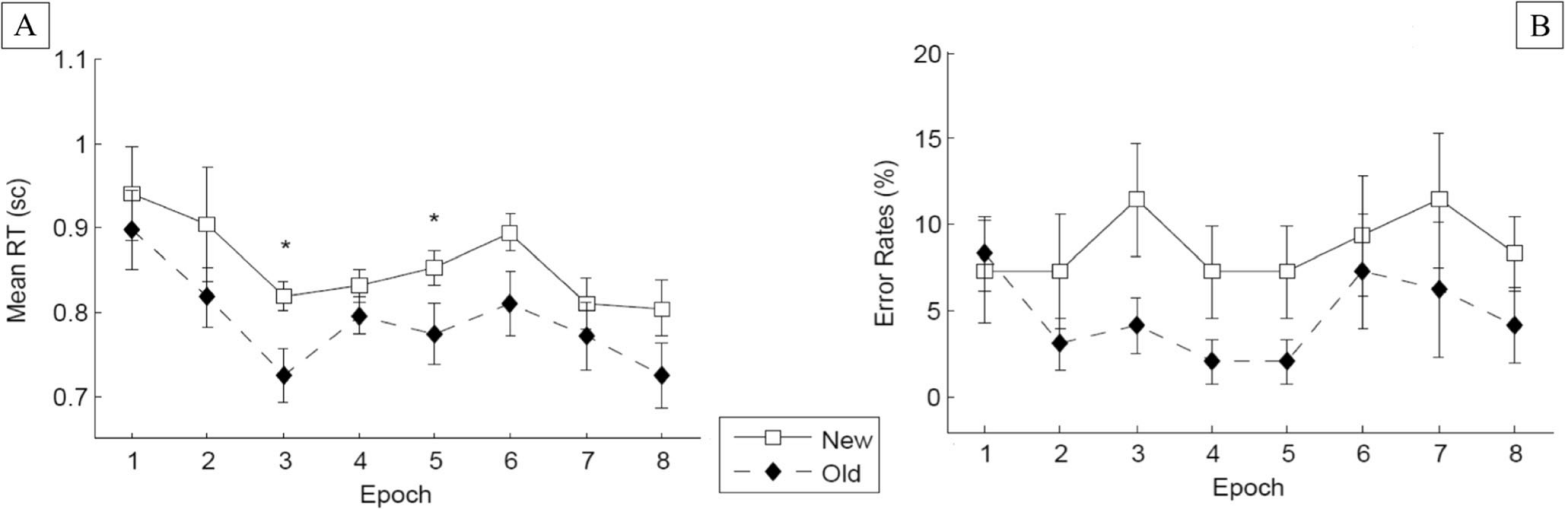
Experiment 2: Mean response times (**a**) and error rates (**b**). See Fig. 3 for information about the error bars. ^*^ *p* < .05

A 2 × 8 factorial repeated measures ANOVA on the RTs revealed a significant main effect of configuration, *F*(1, 11) = 6.02, *p* < .05, *η*_p_^2^ = .354: Targets embedded in old tactile arrangements were responded to faster than targets in new configurations (775 vs. 885 ms), resulting in a contextual-cueing effect of 110 ms. No other effects reached statistical significance [epoch, *F*(7, 77) = 2.24, *p* = .14; Configuration × Epoch interaction, *F*(7, 77) = 0.853, *p* = .547]. Regarding the nonsignificant interaction, additional *t* tests showed no RT difference between old and new configurations in Epochs 1 and 2 (*p* > .1; see Fig. 4). This outcome suggests that, as in Experiment 1, tactile contextual cueing developed as the experiment progressed.

#### Error analysis

As in Experiment 1, a 2 × 8 factorial repeated measures ANOVA on response accuracy failed to reveal a significant effect of configuration, *F*(1, 11) = 3.32, *p* = .09: Given that participants again made fewer errors when searching for targets in old (2.34 %) than in new (4.36 %) configurations, a further *t* test comparing the RTs on error trials between old and new configurations was performed. Once again, the *t* test failed to reveal a significant effect of configuration, *t*(8) = 1.75, *p* = .117, again ruling out a speed–accuracy trade-off. No further effect reached statistical significance [epoch, *F*(7, 77) = 0.662, *p* = .703; Configuration × Epoch interaction, *F*(7, 77) = 0.581, *p* = .769].

#### Recognition performance

Recognition accuracy was assessed in terms of the signal-detection-theoretic measure *d'* (Green & Swets, 1966). For each participant, *d'* was computed, taking into account participants’ hit rates (correct judgments of old configuration as repeated) and false alarm rates (incorrect judgment of new configuration as repeated). An explicit effect would be indicated by *d'* being significantly greater than zero. However, across all participants, *d'* was quite low (0.314) and statistically indistinguishable from zero, *t*(11) = 1.81, *p* = .09, suggesting that tactile contextual cueing is an implicit effect.

### Discussion

Experiment 2 replicated the tactile contextual-cueing effect found in Experiment 1, thus corroborating the idea that participants can learn repeated target–distractor arrangements in tactile search. Interestingly, the extensive practice reduced drastically the response errors in the experimental session (Exp. 2 vs. Exp. 1: old, 2.34 % vs. 7.20 %; new, 4.36 % vs. 13.80 %). Notably however, even after such a marked reduction of response errors, fewer error responses were still made to targets presented in old than in new arrays. Finally, and of the greatest importance, the results of the recognition test suggested that memory for old configurations is implicit, since participants were unable to tell apart old from new configurations.

One objection to Experiment 2 (and Exp. 1) may have been that foot responses were always congruent with the target hand. That is, after detecting an odd-one-out tactile stimulus at the fingers of a given hand (i.e., the target), it is conceivable that the corresponding foot pedal could be pressed “automatically.” In other words, the RT benefit for old relative to new tactile arrangements might reflect context-based facilitation of stimulus- (i.e., hand-) to-response mapping (for the sake of simplicity, we will refer to this as the “response hypothesis”), rather than, or in addition to, contextual cueing of target selection (the “attention hypothesis”). To disentangle these alternative hypotheses, Experiment 3 introduced a discrimination task (as opposed to the localization task in Exps. 1 and 2), in which participants had to first detect and subsequently discriminate the waveform of the target signal. That is, the foot pedals were associated with the target waveform, rather than with the target hand. Under these conditions, the response hypothesis would predict no RT advantage for old relative to new tactile contexts, whereas the attention hypothesis would still predict a benefit for old arrangements.

## Experiment 3

### Method

Experiment 3 was similar to Experiments 1 and 2, except that it used a discrimination task. Furthermore, a new practice session was implemented in order to familiarize participants with the two different target signals.

#### Participants

A group of 14 new participants took part in Experiment 3 (nine female, five male, 12 right-handed; age range 21 to 30 years). The criteria for participation, payment, and ethical guidelines were the same as in Experiments 1 and 2. Of all participants, only one did not provide data for the recognition test, owing to technical issues. The percentage of trials excluded due to incorrect responses was 9.3 %.

#### Apparatus and stimuli

**Fig. 5:**
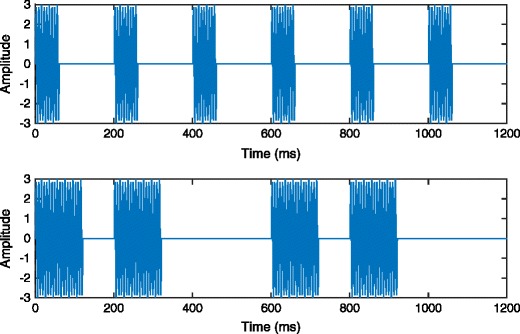
Waveforms of two tactile targets. The upper panel indicates the waveform of Target 1 (T1), a 5-Hz square wave with a 30 % duty cycle delivered via 150-Hz vibrations. The lower panel shows the waveform of Target 2 (T2), a burst square wave (mean frequency of 4.17 Hz) with an average 30 % duty cycle delivered via 150-Hz vibrations. The distractors are constant vibrations of 150 Hz

In order to create (three) distinct signals for targets (two signals) and distractors (one signal), the solenoid actuators were controlled by a new, 10-channel Tactor Amplifier (Dancer Design) connected to a standard PC equipped with a National Instrument Card (NI PXI-1042Q). The two possible tactile targets, T1 and T2, were defined by a square waveform manipulation of 150-Hz vibrations (Fig. 5); the distractors, by contrast, were constant 150-Hz vibrations.

#### Procedure

The practice session was divided into two parts. In the first part, participants learned the identities of the two possible targets. One of the targets was presented in isolation per trial in a randomized fashion on each finger of each hand (except the thumbs). Furthermore, because the features of each target were rather technical for written or verbal instructions, in the first 16 trials of the practice participants received a visual cue informing them of the identity of the current target “T1” or “T2,” so they could learn the physical properties and the appropriate foot response. In the second half of the practice session, participants trained on the tactile search task with one target and seven distractors (similar to Exps. 1 and 2; no visual cues as to the target identity were given). Participants were instructed to respond as quickly and accurately as possible, within 3,000 ms. They received “error warning” beeps and the intertrial intervals after erroneous responses. Of note, progress from the first to the second part of the practice session and from the practice to the test session was only possible when participants achieved 80 % accuracy in each of the two practice phases. Target pedal assignments were counterbalanced across participants: Half of participants used the left (vs. right) foot pedal for “T1” (vs. “T2”), and vice versa for the other half. The entire experimental session lasted 30–50 min, depending on participants’ performance in the training session.

#### Recognition task

At the end of Experiment 3, participants performed a “yes–no” recognition test similar to that in Experiment 2.

### Results

#### RT performance

**Fig. 6:**
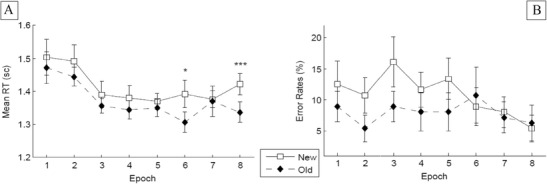
Experiment 3: Mean response times (**a**), and error rates (**b**). See Fig. 3 for information about the error bars. ^*^ *p* < .05, ^***^ *p* < .001

A 2 × 8 factorial repeated measures ANOVA on the RTs revealed a significant main effect of configuration, *F*(1, 13) = 5.95, *p* < .05, *η*_p_^2^ = .314: Discrimination was faster for targets embedded in old as compared to new tactile configurations (1,373 vs. 1,416 ms), indicative of a tactile contextual-cueing effect (of 43 ms). No further effect was significant [epoch, *F*(7, 91) = 2.55, *p* = .08; Configuration × Epoch interaction, *F*(7, 91) = .567, *p* = .781]. Regarding the nonsignificant interaction, additional *t* tests revealed that the RT difference between old and new configurations became significant only late in the experiment, at Epoch 6 (*p* < .05; see Fig. 6).

#### Error analysis

A 2 × 8 factorial repeated measures ANOVA on response accuracy failed to reveal a significant effect of configuration, *F*(1, 13) = 2.49, *p* = .138. However, because participants made fewer errors when searching in old (3.9 %) than in new (6.36 %) configurations, a further *t* test comparing the error RTs between the two conditions was performed. The analysis failed to reveal a significant effect of configuration, *t*(11) = 1.61, *p* = .134, once again ruling out a speed–accuracy trade-off in the determination of the RT results. No further effect was statistically significant [epoch, *F*(7, 91) = 0.139, *p* = .251; Configuration × Epoch interaction, *F*(7, 91) = 0.681, *p* = .688].

#### Recognition performance

Across all participants, *d'* was quite low (–0.17) and statistically indistinguishable from zero, *t*(13) = –1.29, *p* = .218. This result further supports the findings of Experiment 2—namely, that tactile contextual cueing is mediated by implicit memory representations.

### Discussion

In Experiment 3, we examined whether RT benefits for old versus new tactile configurations were due to facilitated (learned) stimulus-to-response mappings or facilitated target selection. Employing a target discrimination task—in which different vibrotactile stimuli (T1 and T2), each of which could occur in either hand, were mapped to the foot responses—RTs were found to be still faster for old tactile arrangements. This largely rules out the possibility that the reduced RTs for repeated tactile-search arrangements are attributable to facilitation of response selection. Instead, the finding of an RT benefit for old arrangements strongly supports the alternative view of tactile contextual cueing facilitating attentional target selection (rather than postselective stimulus-to-response mapping). The analysis of error rates revealed no significant differences between old and new configurations. However, as in Experiments 1 and 2, if anything, there was a (numerical) accuracy advantage for old over new tactile arrangements in Experiment 3—that is, here, greater accuracy in discriminating, rather than localizing, the target in old configurations. This further supports the idea that configural learning did aid attentional target selection, and thus subsequent (postselective) processes of focal-attentional target discrimination. Finally, the results of the recognition test corroborate the idea that tactile contextual cueing is supported by an implicit memory, since participants were not able to tell apart old from new configurations.

It should be noted that the results of Experiment 3 do not rule out potential contributions of response selection to the RT advantages for old arrangements in Experiments 1 and 2. In fact, in Experiments 1 and 2, contextual cueing was twice as large as in Experiment 3 (103, 110, and 43 ms in Exps. 1, 2, and 3, respectively). It may well be that the reduction of the effect in Experiment 3 reflects the fact that the perfect coupling of hands and foot pedals (and learning of the couplings for repeated arrangements) in the previous experiments contributed to the overall RT advantage for old arrangements. Given that such a contribution was effectively ruled out in Experiment 3 (by making the coupling inconsistent), the tactile “contextual-cueing” effects in Experiments 1 and 2 are likely to represent an additive mixture of both facilitation of target selection and facilitation of response selection. This “hybrid view” would suggest that contextual cueing can exert a boosting influence on both target and response selection, consistent with Kunar, Flusberg, Horowitz, and Wolfe (2007), who argued for such a view in relation to visual contextual cueing.

Note that, in the present study, we inferred the effect of contextual cueing on attentional selection only indirectly, by comparing the effects of old versus new configurations between discrimination and a localization task. Thus, ideally, this evidence should be followed up in a more direct test, involving a set size manipulation or a direct measure of the brain-electrical (electroencephalographic) activity indexing the allocation of attention. Such direct tests are, however, beyond the scope of the present study, especially since they would introduce new challenges, such as whether a set size manipulation is an appropriate means for inferring attentional guidance by contextual cueing (see, e.g., Kunar et al., 2007, and Kunar, Flusberg, & Wolfe, 2008, for discussions), or which brain region provides an apt electrophysiological signal for context-based guidance in tactile search (one candidate area is the somatosensory cortex; cf. Eimer, Maravita, Van Velzen, Husain, & Driver, 2002). In the meantime, though, the important observation remains that tactile contextual cueing was reliable in a discrimination task in which there was no consistent hand- (configural-pattern-) to-foot mapping. This strongly suggests that response selection cannot be the sole source of the contextual-cueing effect. Instead, the effect also involves a component of attentional guidance.

## General discussion

In the present study, we aimed at answering two questions: First, can contextual cueing arise from repeated exposure to purely tactile search configurations? And second, is tactile contextual cueing an implicit effect? Three experiments were conducted to answer these questions. The aim of Experiment 1 was to test whether contextual cueing, an effect hitherto examined almost exclusively in the visual domain, would also operate in tactile search. Experiment 2 was, additionally, designed to assess participants’ explicit knowledge of the repeated tactile configurations. And in Experiment 3 we aimed at dissociating the effects of old (vs. new) tactile arrangements on attention and response selection. The results were as follows: Response speed and accuracy were improved for old relative to new configurations in all three experiments, indicating that memory for repeated tactile configurations is acquired and subsequently expressed in tactile search. Furthermore, participants’ ability to distinguish the old from the new configurations was only at chance level, indicative of tactile contextual cueing being supported by an implicit memory system (Exps. 2 and 3). Moreover, contextual cueing was observed to be sufficiently strong to aid performance even in a target discrimination (rather than only in a target localization) task (Exp. 3). Taken together, these findings show that the tactile system is able to develop its own context representations and use these representations to guide tactile search. Furthermore, the build-up of memory for repeated tactile configurations is an automatic process, in that it does not require explicit knowledge of any repeated configurations.

To our knowledge, the present findings are the first to show that invariant spatial configurations presented exclusively to the tactile modality can be learned and can subsequently facilitate tactile search. To date, only one study has demonstrated effects of learned configurations (context) in tactile search. According to Nabeta et al. (2003), contextual knowledge acquired solely within the visual modality can subsequently facilitate haptic search. However, as we pointed out in the introduction, the results of Nabeta et al. are open to alternative interpretations, such as that haptic contextual cueing is mediated by a visuospatial representation that may trigger learned visual context associations to guide haptic search. Most importantly, Nabeta et al. did not directly address the issue of whether haptic contextual cues can be learned when repeated target–distractor configurations are presented exclusively to the haptic modality. Concerning this issue, our findings unequivocally show that contextual cueing develops when sighted, but blindfolded, participants are required to discriminate the location or identity of a feature singleton target in tactile search.

With the present design endeavored to implement the essential features of the visual contextual-cueing paradigm—in particular, the presentation of repeated configurations on half of the trials (Chun, 2000; Chun & Jiang, 1998; Conci et al., 2013; Geyer et al., 2013; van Asselen & Castelo-Branco, 2009). Nevertheless, some limitations prevent a direct comparison between the present tactile and the “standard” visual paradigm. In visual contextual cueing, items are distributed across a relatively large display area with a large number of possible locations—for example, 6 × 8 locations in Chun and Jiang’s study. Moreover, a typical configuration consists of 12 items, one of which is the target “T,” and the 11 others, distractor “L”s. A few studies have also used smaller set sizes of four items or larger sizes of 16 items, or have manipulated set size actively in an attempt to investigate the effects of contextual cueing on the efficiency of attentional selection (e.g., Annac et al., 2013; Chun & Jiang, 1998; Kunar et al., 2008; Makovski & Jiang, 2010). Accordingly, the number of possible item configurations is quite large, ranging up to thousands of configurations in a given experiment.

In the present, tactile version of the contextual-cueing paradigm, by contrast, a manipulation of set size was not possible; instead, this was fixed at four items, comprising one target and three distractors on each trial. The set size and item features were tightly restricted, owing to a few crucial reasons, including the limited number of possible item locations (eight fingers), low vibrotactile discrimination sensitivity (Lederman & Klatzky, 2009), and device limitations. Due to those limiting factors, the number of possible tactile configurations was reduced dramatically. In an attempt to make the paradigm more similar to visual contextual cueing, the number of trials in tactile search could, in principle, be increased by producing different vibrotactile patterns to generate different distractors (but, nevertheless, in the same configurations). Arguably, however, this would come at the cost of making the task considerably more difficult to perform. Recall that in the present setup, although the target was a feature singleton (rather than a conjunction target, as in the standard visual paradigm), response errors occurred on a high proportion of trials, even after participants had extensively practiced the task.

Finally, again considering the limitations of implementing a practicable tactile contextual-cueing paradigm, some of the results lacked statistical power, particularly those of the recognition tests: Recall that the test comprised only 16 trials. However, this argument would also apply to almost all visual contextual-cueing studies, since they typically use (visual) tests that are rather short (even though there are no obstacles to conducting longer tests; see, e.g., Schlagbauer et al., 2012, and Smyth & Shanks, 2008, including their discussion of power problems in explicit tests of contextual cueing). Central to the limitation in the present type of (tactile) setup in tactile search is again the set size problem, which should be addressed in the future.

Despite of the limitations above, our results provide an initial understanding of the independent capacity of the tactile modality that can permit invariant spatial configurations to be extracted and stored in terms of long-term memory representation that may be activated by currently encountered tactile configurations, and thus guide tactile search—quite similar to visual contextual cueing (Chun & Jiang, 1998). Consequently, this demonstration lends itself to addressing a number of new important questions concerning tactile contextual cueing. In particular, with reference to cross-modal cognitive processes, it would be of prime importance to investigate whether spatial regularities learned in the tactile modality could also facilitate visual search, and vice versa. Due to the limited evidence that is available on cross-modal implicit spatial learning, it is not yet clear whether configural representations that are formed in specific modalities are also shared across multiple—namely, visual and tactile—sensory domains. For example, Newell, Woods, Mernagh, and Bülthoff (2005) found that both visual and haptic representations (of seven wooden animals presented in front of the participant) were dependent on the position of the participant through encoding, suggesting that both representations are supported by a viewer-centered reference frame. However, when participants had to judge which two of seven objects were in new positions, accuracy performance was higher in the intramodal (e.g., visual–visual) than in the cross-modal (e.g., haptic–visual) conditions. Note that in Newell et al.’s study, the experiments were divided into a training phase (intended for the acquisition of either a visual or a haptic representation) and test phase (intended for the expression of these representations). Their results suggest that encoded representations contain not only information about the orientation of the scene, but also about the encoding modality. That is, objects experienced visually or haptically in an explicit learning task form their own spatial representations. The results of the present experiments support the notion that (tactile) contextual cueing can originate from repeated encounters with tactile search configurations, making the question of modality-dependent versus -independent memory representations in contextual cueing, and in implicit perceptual learning in general, an interesting issue for future investigations.

In summary, the present study has shown that tactile configural regularities can be learned and can subsequently guide tactile search, and that this process is rendered implicitly. This finding provides the first evidence that powerful “implicit memory” mechanisms allow specific tactile information to be retained from the sensory environment and to persist over time. These implicit memory traces contribute to the guidance of attention in the real-time processing of the perceptual array, making processing efficient by reducing the need for capacity-limited, top-down-controlled mechanisms.

## Footnotes

1 In a control analysis, we examined the variability in distractor positions, measured as the standard error (*SE*) of the RTs in old and new configurations. It was possible that at least parts of the RT difference between the two types of configurations could be attributed to greater variability in distractor positions in the new configurations (in addition to contextual cueing of haptic search in the old configurations). However, a *t* test comparing the *SE*s between the new and old configurations was nonsignificant: *t*(8) = 1.39, *p* = .202. Similar results were obtained for Experiment 2 [*t*(11) = 0.000, *p* = 1.00] and Experiment 3 [*t*(13) = 1.52, *p* = .15]. This ruled out the alternative account.
